# Non-surgical oral hygiene interventions on disease activity of Rheumatoid arthritis patients with periodontitis: A randomized controlled trial

**DOI:** 10.34172/joddd.2020.004

**Published:** 2020

**Authors:** William Buwembo, Ian Guyton Munabi, Mark Kaddumukasa, Haruna Kiryowa, Muhammad Mbabali, Ethel Nankya, William Evan Johnson, Emmy Okello, Nelson K. Sewankambo

**Affiliations:** ^1^Department of Human Anatomy, School of Biomedical Sciences, Makerere University College of Health Sciences, Kampala Uganda; ^2^Department of Medicine, School of Medicine, Makerere University College of Health Sciences, Kampala Uganda; ^3^Department of Dentistry, School of Health Sciences, Makerere University College of Health Sciences, Kampala Uganda; ^4^Division of Computational Biomedicine, Boston University School of Medicine, Boston, MA, Boston University, USA

**Keywords:** DAS-28 score, periodontitis, periodontopathogenic bacteria, pocket depth, rheumatoid arthritis

## Abstract

***Background.*** Periodontitis and rheumatoid arthritis have similar epidemiology and pathophysiology. Understanding the interaction between these two diseases is vital in our settings. We set out to assess the effect of oral hygiene interventions on disease activity of rheumatoid arthritis patients with periodontitis in Kampala, Uganda.

***Methods.*** Fifty-eight patients attending an arthritis clinic with rheumatoid arthritis and periodontitis were randomly assigned to either an intervention group or a control group. Patients diagnosed with rheumatoid arthritis at least two years before, who were on the same medication, dose, or formulation for RA treatment during the preceding three months, were included. The patients were >18 years of age, would be available for all the study visits in the next six months, had at least six natural teeth, had periodontal disease classified as Dutch Periodontal Index (DPSI) >3 and provided written informed consent. Those who had a chronic disorder requiring chronic or intermittent use of antibiotics, were pregnant, were lactating, or had intent to become pregnant were excluded. The primary outcome measure was a change in Disease Activity Score of 28 Joints (DAS28 score) in two 3-month follow-up periods after the intervention. The secondary outcome measure was a change in periodontal status.

***Results.*** There was a statistically significant improvement in the DAS-28 score in both the intervention and control arms during the follow-up period (P<0.01). The participants carrying more than one bacterial species had worse DAS-28 scores.

***Conclusion.*** Oral hygiene interventions given to RA patients could drastically improve their RA treatment outcomes, especially in resource-limited settings.

## Introduction

Rheumatoid arthritis (RA) is a chronic systemic inflammatory autoimmune disease of the connective tissue that predominantly affects the synovial membranes of diarthrodial joints, characterized by joint swelling, joint tenderness, and destruction of synovial joints, leading to severe disability and premature mortality.^1-4^ Periodontitis (PD) is an inflammatory, infectious disease, resulting in the destruction of tooth-supporting tissues, eventually leading to tooth loss.^5^ RA and PD are chronic inflammatory diseases, strongly associated in epidemiology and pathophysiology, affecting humans worldwide.^6^

The association between RA and PD is thought to be through periodontopathogenic bacteria of the red complex, i.e., *P. gingivalis , A. actinomycetemcomitans , F. nucleatum*, and *T. forsythia*. Among these periodontopathogens, *P. gingivalis* and *A. actinomycetemcomitans* have been linked to anti-citrullinated protein antibodies (ACPAs) in patients with RA.^7^ The *P. gingivalis* bacteria produce citrullinated proteins with the aid of peptidylarginine-deiminases (PADs), enzymes that catalyze the conversion of peptidylarginine sections of proteins to peptidyl-citrulline. This citrullination leads to the loss of tolerance to neo-epitopes, eliciting a response that might result in RA.^8^ While *A. actinomycetemcomitans* produces a toxin (leukotoxin A, LtxA) that triggers global hypercitrullination in neutrophils, and has also been recently linked to rheumatoid arthritis (RA) pathogenesis.^9^ In a recent meta-analysis, it was observed that compared to the general population, subjects with RA are at an increased risk of developing PD, and vice versa (relative risk: 1.13; 95% CI: 1.04, 1.23; P=0.006; N = 153,277).^10^ The clinical course of PD in RA patients is more severe compared to non-RA individuals.^6^ Together, the development of both diseases brings considerable consequences for public health and the quality of life of the affected individuals. Additionally, RA patients with PD receiving non-surgical periodontal treatment have been shown to have significant improvements in the clinical outcome for RA in studies in developed countries in particular.^11-14^

There is currently limited data from developing countries,^15-17^ with no published information looking at the role of PD in Ugandans with RA. Understanding the burden of PD and designing interventions for our population is important since studies carried out elsewhere have shown that eradication/control of PD results in beneficial reductions in RA disease activity and severity.^18^ Given the higher bacterial disease burden and concurrent low levels of dental care/hygiene that characterizes the population in this region,^19,20^ there is an urgent need to evaluate the effect of oral hygiene measures for periodontal treatment for PD in RA patients. Therefore, the current study aimed to assess the effect of oral hygiene intervention on disease activity of rheumatoid arthritis patients with periodontitis in Kampala, Uganda.

## Methods

### Study design and participants

This was an unmatched open-label randomized control trial in a ratio of 1:1.

### Study site

The study was conducted at the Mulago public national referral and teaching hospital arthritis outpatient clinic at Kiruddu, Kampala, Uganda. The clinic runs once a week, reviews an average of 100 rheumatoid arthritis patients every month. Approximately 40% of the attending individuals have rheumatoid arthritis and usually come to the clinic for patient problems, drug refills, and drug toxicity monitoring, monthly to quarterly. These patients are referred from various clinics and hospitals all over the country. The oral health of these patients is currently not routinely observed as part of their clinical care.

**Figure 1:**
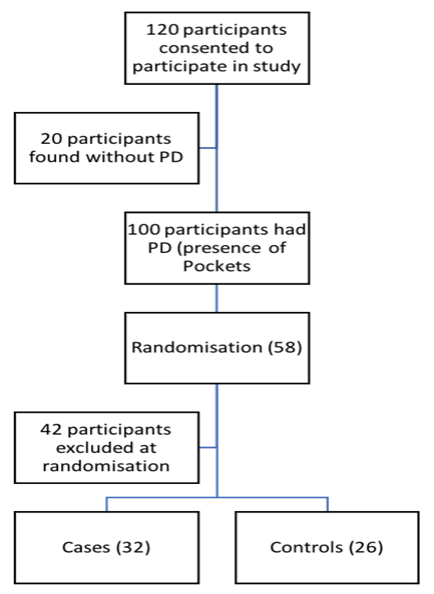
Participant flow diagram To randomise the participants into intervention and control arm, 120 participants with rheumatoid arthritis were recruited. Of the 120 participants, 100 also had periodontitis and were screened for potential enrolment into the study. Of the 100, 58 participants met the study inclusion criteria and provided written informed consent. They were randomly assigned to either an intervention group or to a control group using computer generated assignment random numbers. We excluded 42 study participants who had coexisting known chronic disorders requiring chronic or intermittent use of antibiotics (26), and reported to be pregnant or lactating (12) or intending to become pregnant within the study period (four).

The sample size was estimated at 60 subjects using an online sample size calculator for repeated-measures Rmass^21^ (http://www.rmass.org/), assuming a minimum difference in the DAS28 scores of 0.6^22^ with α=0.05 and β=0.2. An estimated 15% for loss to follow up was included to give the final sample size of 30 individuals per group for intervention and control group (Figure 1).

### Participant screening and enrolment

One hundred patients with confirmed rheumatoid arthritis and periodontitis were screened for potential enrolment in the study; of these, 58 participants met the study inclusion criteria and provided written informed consent to be randomly assigned to either an intervention group or a control group using computer-generated assignment random numbers. The inclusion criteria for this study were;

1. Aged ≥18 years (of either gender)
2. Diagnosed with rheumatoid arthritis at least two years before as classified by the American College of Rheumatology/European League Against Rheumatism (ACR/EULAR)^23^
3. Receiving the same medication, dose, or formulation for RA treatment during the preceding three months before the study
4. Availability to conduct all the study visits over the six-month follow-up
5. Having at least six natural teeth,
6. Provision of informed consent
7. Having periodontal disease classified as Dutch Periodontal Screening Index (DPSI) ≥3.^24^

We excluded 42 study participants who had coexisting known chronic disorders and required chronic or intermittent use of antibiotics, and those who reported to be pregnant or lactating or intending to become pregnant within the study period.^25^

The participants underwent initial examinations at the beginning of the trial and underwent either non-surgical periodontal treatment (scaling, polishing root planing) within three weeks after the first visit or given oral hygiene instructions for use at home. Both study group subjects received a mouthwash, CitrollinÒ (containing cetrimide and lidocaine HCl, from Pharco Pharmaceuticals, Alexandria, Egypt), and were advised to use 10 mL of the mouthwash twice a day after toothbrushing for 10 days after the OHI session. Both groups were followed up at three months and six months. During these visits, the DAS-28 scores and periodontal condition were re-assessed; the patients continued to attend the rheumatology clinic according to their schedules. At all these visits, subgingival plaque samples were picked from the participants who had a pocket depth of DPSI >3.

A focused clinical examination was conducted by a rheumatologist (MK), and disease activity was assessed using the DAS-28 score.^26^ An oral examination was performed by the same dentist (HK) to assess the visible plaque index,^27^ gingival bleeding index,^28^ and pocket depth, based on the Dutch Periodontal Screening Index (DPSI) using a manual periodontal color-coded standard probe (Dentsply™, London, UK).

Oral hygiene instructions (OHI) were given by dental investigators (WB, MM, and HK). This was a 15-minute oral session with visual and verbal information on how to use a toothbrush, dental floss, and mouthwash.

10 mL of blood sample was collected by venipuncture from each participant. Sera were analyzed for ESR, which is carried out routinely at Mulago Hospital lab and was used to calculate the DAS-28 score. The subgingival plaque was harvested by individually packed toothpicks sterilized by autoclaving from the six most periodontally diseased sites of all the participants. The plaque samples were pooled to extract DNA, using the Promega Wizard™ Genomic DNA Purification Kit, according to the manufactures’ instructions. Extracted DNA was used to determine the presence of periodontal pathogens by PCR.^29,30^ PCR amplification was performed in volumes of 25 μL containing 1X PCR reaction buffer/Mg^++^ (Bioland Scientific LLC, CA, USA), 0.2 mM of dNTP (Bioland Scientific LLC, CA, USA), 0.5 U of *Taq* DNA polymerase (Bioland Scientific LLC, CA, USA), 10 ng of template and 0.2 μM of each primer pairs, i.e. *A. actinomycetemcomitans*: GCT AAT ACC GCG TAG AGT CGG & ATT TCA CAC CTC ACT TAA AGG T; *T. forsythia*: GCG TAT GTA ACC TGC CCG CA & TGC TTC AGT GTC AGT TAT ACC T; *F. nucleatum*: ATT GTG GCT AAA AAT TAT AGT T & ACC CTC ACT TTG AGG ATT ATA G; and *P. gingivalis*: AGG CAG CTT GCC ATA CTG CG & ACT GTT AGC AAC TAC CGA TGT. Amplification was performed in a SimpliAmp^TM^ from Thermal Cycler Thermo Fisher Scientific (Applied Biosystems, MA, USA) programmed for 94ºC(2 minutes), followed by 30 cycles at 94ºC (0.5 minutes), adequate annealing temperature for each primer pair: *A. actinomycetemcomitans*: 50ºC and amplicon length of 0.5 Kb; *T. forsythia*: 60ºC and amplicon length of 0.6 Kb; *F. nucleatum*: 50ºC and amplicon length of 1 Kb; and *P. gingivalis*: 60ºC and amplicon length of 0.4 Kb, extension of 72ºC (1 minute), followed by 72ºC (5 minutes) to allow the completion of DNA extension. A negative control without template DNA was included in each PCR run. The amplification products were compared by electrophoresis in 2% agarose gel (in 1X TBE [1 M Tris, 0.9 M boric acid, 0.01 M EDTA, pH 8.4]) buffer (VWR International GmbH - Darmstadt, Germany), stained with ethidium bromide (0.5 μg/mL), and photographed on a UV light transilluminator (Kodak Digital Science System 120). Molecular mass standard 100-bp ladder (New England Bio Labs) was included.

### Data analysis

Data were analyzed using STATA version 15 by various panel data approaches to compare the multiple visit observations on each participant with the level of significance set at 0.05. The panel data approaches used included: multilevel linear regression modeling with additional maximum likelihood imputations following an intention to treat.^31^ In addition, panel data time to event analysis was used to compute the hazard risk ratio for finding any one of the tested bacteria in the sample of subgingival plaque from the study participants and multinomial regression to compare participants with different levels of clinical Das-28 score improvement. The data for the time to event and multinomial analysis was obtained from the differences in the observations made at the different study clinic visits. The computation of the DAS-28 score improvement was made following the procedure outlined in the clinical guidelines by The European League Against Rheumatism response criteria.^32^ We reported on the various descriptive statistics for the different proportions and/or mean outcomes, hazard ratios,^33^ and relative risks of the associated study measures. The Venn diagram was generated using the Limma package^34^ of the R statistical computing environment.^35^

The study had ethical clearance by the Makerere University School of Biomedical Science (protocol ID SBS 457) Institutional Review Board, the Uganda National Council of Science and Technology (HS 2287) and was registered as a clinical trial (clinical trials.gov ID NCT03513263). Written informed consent was obtained from all the participants, and the standard of care was followed.

## Results

A total of 58 participants were enrolled in this study (Figure 1, the participant flow diagram). Fifty-five% (32/58) of the subjects were in the intervention arm of the study, while the remaining 26/58 (44.8%) were in the control arm. Both recruitment and eventual follow-up of the participants for this trial was carried out from September 2017 to December 2018. Four study participants in the control arm were lost for follow-ups after the first visit. The overall mean age of the study participants was 50.2 years; the majority of the study respondents were female. Table 1 provides a summary of the characteristics of all the participants at baseline. Table 2 shows no differences between the intervention and control arms for the various measures in the study.

**Table 1:** Descriptive baseline statistics

**Marital status**	**Number (percentage)**
Divorced	1 (1.92)
Married	35 (67.31)
Single	16 (30.77)
**Residence**	**Number (percentage)**
Urban	31 (54.39)
Peri-urban	13 (22.81)
Rural	13 (22.81)
**Level of education**	**Number (percentage)**
Primary education	16 (33.33)
Secondary education	24 (50.00)
University education	8 (16.62)
**Employment status**	**Number (percentage)**
Unemployed	15 (28.85)
Employed	10 (19.23)
Self employed	22 (42.31)
Retired	5 (9.62)
**Sex**	**Number (percentage)**
Male	10 (17.24)
Female	48 (82.76)
	**Median (N, IQR)**
**Age**	50.5 (58, 40 to 61)
**Pocket depth**	3 (58, 3 to 4)
**Plaque score**	0.67 (58, 0.17 to 1.17)
**Gingival score**	0.33 (58, 0.08 to 0.83)
**Days between visits 1 and 2**	95 (176, 70 to 143.5)
**Days between visits 2 and 3**	105 (112, 91 to 157.5)
**DAS-28 score**	6.75 (55, 4.94 to 7.40)

**Table 2:** Differences in the baseline study variables relative to randomization

**Variable**	**Control**	**Intervention**	
	**Median (N, IQR)**	**Median (N, IQR)**	**z-score (P-value)**
Marital status	2 (24, 2 to 3)	2 (28, 2 to 2)	1.69 (0.09)
Residence	0 (25, 0 to 2)	1 (32, 0 to 2.5)	-0.71 (0.48)
Level of education	1 (23, 0 to 1)	1 (25, 0 to 1)	0.18 (0.24)
Employment status	1.5 (24, 0.5 to 2)	2 (28, 0 to 2)	0.26 (0.79)
Sex	1 (26, 1 to 1)	1 (32, 1 to 1)	-1.05 (0.29)
	**Mean (N, SD)**	**Mean (N, SD)**	**t-score (p-value)**
Age	49.04 (26, 13.71)	51.09 (32, 14.06)	-0.56 (0.58)
ESR	52.16 (25, 32.99)	40.43 (30, 21.95)	1.57 (0.12)
Swollen joints	12.73 (26, 9.95)	10.40 (30, 10.18)	0.86 (0.39)
Painful joints	17.65 (26, 8.13)	15.73 (30, 11.85)	0.76 (0.45)
Pocket depth	3.27 (26, 0.45)	3.31 (32, 0.54)	-0.33 (0.74)
Plaque score	0.75 (26, 0.51)	0.84 (32, 0.52)	0.75 (0.46)
Gingival score	0.50 (26, 0.51)	0.51 (32, 0.51)	0.09 (0.93)
Reported global score	5.5 (26, 1.91)	5.93 (32, 1.91)	-0.90 (0.37)
Days between visits 1 and 2	133.5 (80, 96.99)	128.58 (96, 90.55)	0.34 (0.74)
Days between visits 2 and 3	115.5 (48, 48.92)	138.19 (64, 69.20)	-1.94, (0.06)
Das score	6.28 (26, 1.58)	5.91 (29, 1.54)	0.88 (0,38)

### Rheumatoid arthritis disease activity

At baseline, 5.4% (3/56) of the subjects had low disease activity scores (DAS-28) ranging from 2.6 to 3.2, while 19.6% (11/56) had a moderate disease activity score range of 3.2–5.1. The majority (42/56 [75%]) had severe disease activity scores of >5.1. When compared with the controls, the participants in the intervention arm were 0.42 times more likely to have moderate baseline disease activity scores relative to the participants with low disease activity scores (95% CI: 0.03‒6.06, P=0.52). The subjects in the intervention arm were also 0.60 times (95% CI: 0.05‒7.19, P=0.691), more likely to have severe baseline disease activity scores relative to the participants with low disease activity scores in the control arm.

**Figure 2:**
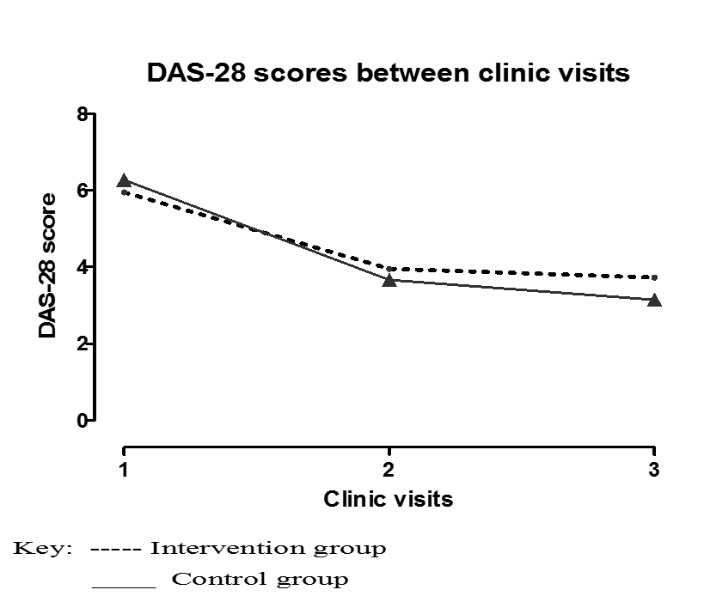
Comparing the DAS-28 scores between three clinical visits over 6 months. To assess the effect of oral hygiene interventions on disease activity of Rheumatoid arthritis patients with periodontitis in Kampala, Uganda. Participants were randomised into an Intervention and a control treatment groups. These were followed for two 3 months periods. Overall, both groups showed remarkable reductions in their DAS-28 scores from baseline which was more marked in the first 3 months.

Table 3 provides a summary of the follow-up observations for the study participants. The DAS-28 scores that were the primary outcome of the study exhibited an improvement (a change in DAS-28 score >1.2) for 21/75 (28.00%) of the participants. There were also 24/75 (32.00%) examinations where a moderate improvement (a change in DAS-28 score <0.6 but ≥1.2) was observed and other 30/75 (40.00%) examinations where there was no clinical improvement (a change in DAS-28 score ≤0.6). As shown in Table 2, most of the good-to-moderate improvement was seen after the first review, while no improvement dominated the examinations at the second review visits. This difference in the visit observations was significant (chi-squared=15.9, P≤0.01). Overall, there was no difference between the observed improvement in DAS scores between the two arms of the study (RR=1.00 n=42, 95% CI: 0.95‒1.05, P=0.97) (Figure 2).

**Table 3:** Overall summary of the participants’ follow-up review observations

**DAS response on follow up visit**	**Number (percentage)**
No response	30 (40.00)
Moderate response	24 (32.00)
Good response	21 (28.00)
***P. *** ***Gingivalis*** **presence at each visit**	**Number (percentage)**
New infection	2 (4.35)
No change	30 (65.22)
Cured	14 (30.43
***F. *** ***nucleatum*** **presence at each visit**	**Number (percentage)**
New infection	0 (0)
No change	36 (78.26)
Cured	10 (21.74)
***A.*** ***actinomycetemcomitans*** **presence at each visit**	**Number (percentage)**
New infection	3 (6.52)
No change	32 (69.57)
Cured	11 (23.91)
***T. forsythia*** **presence at each visit**	**Number (percentage)**
New infection	3 (6.52)
No change	33 (71.74)
Cured	10 (21.74)

### Periodontopathogenic bacteria

**Figure 3:**
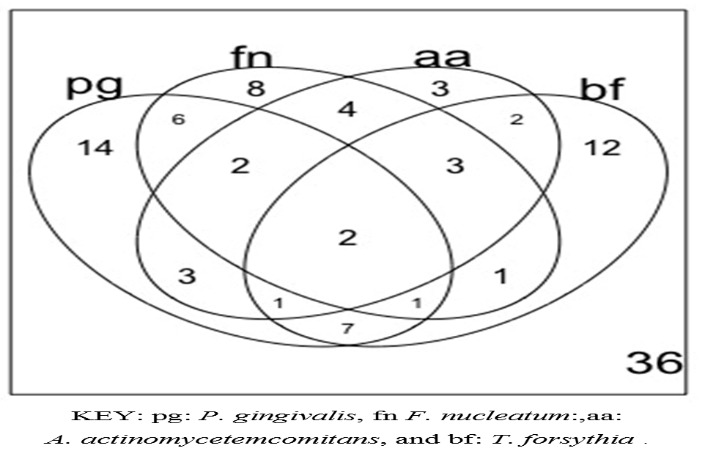
Venn Diagram showing participants occurrence of periodontopathic bacteria To determine the occurrence of the four common periodontopathogenic bacterial species in the collected subgingival plaque at difference oral examination, PCR detection was used. In this diagram note that there were two samples from two oral examinations that had all four bacteria, 14 samples with P.gingivalis only and 36 samples with none of the four bacteria.

The Venn diagram in Figure 3 shows the number of study participants with each bacterial species screened for, identified in each of the corresponding oral examinations associated with a sample collection. At baseline, 18.90% (11/58) of the subjects were found with none of the four bacterial species screened for; 36.20% (21/58) had one of the four bacterial species and 44.80% (26/58 ) had two or more (mixed infection) of the bacteria screened for. During the second visit, this changed to 42.40% (14/33), with none of the bacteria screened for, 39.40% (13/33) with one of the four bacteria, and only 18.20% (6/33) exhibiting two or more bacterial species screened for. In the final visit, the majority of the subjects were found with none of the four bacteria screened for (11/14, 78.60 %), and only three (3/14, 21.40%) with one of the four bacteria screened for. The observed reduction in the variety of the bacterial species screened for was significant (chi-squared=23.7, P<0.01). On average, the DAS-28 scores increased by 0.69 with the presence of any of the four bacteria screened for, which was significant (0.69, 95% CI: 0.37‒1.01, P<0.01, with a constant of 5.23 [(95% CI: 4.76‒5.70, P<0.01]). On average, the DAS-28 scores were also higher for the presence of *F. nucleatum* (0.07, 95% CI: 0.28‒0.42, P=0.71), *A.*  *actinomycetemcomitans* (0.10, 95% CI: -0.25‒0.45, P=0.56) and *T. forsythia* (0.07, 95% CI: -0.28‒0.42, P=0.71), compared with the presence of *P. gingivalis* ; this difference was not significant. Compared to the participants with none of the four bacteria screened for, those with one (1.27, 95% CI: 0.94‒1.61, P=0.01) or two or more (2.31, 95% CI: 1.88‒2.76, P<0.01) of the bacteria screened for had significantly higher DAS-28 scores (constant: 4.27, 95% CI: 3.78‒4.74, P<0.01). This model, showing the effect of the number of bacterial types found on the DAS-28 scores, was significant and accounted for 70% of the variation observed in DAS-28 scores (rho=0.70, 95% CI: 0.60‒0.79, P<0.01). Table 4 shows the changes in the proportions of positive PCR reactions for the selected four bacterial species throughout the three examinations. In this Table, note that most of the mono infections observed by the last visit were due to *P. gingivalis* and *F. nucleatum*.

**Table 4:** Summary of participants’ follow-up examination observation data

	**Number of positive PCR tests (N, %)**			
**Observation**	**Randomization**	**Baseline**	**Visit 2**	**Visit 3**
***P. *** ***gingivalis***	Intervention	18 (32, 56.25)	4 (18, 22.22)	2 (9, 22.22)
	Control	10 (26, 38.46)	1 (17, 5.88)	0 (8, 0.00)
***A.*** ***actinomycetemcomitans*** **infection**	Intervention	8 (32, 25.00)	4 (18, 22.22)	0 (9, 0.00)
	Control	6 (26, 23.08)	2 (17, 11.76)	0 (8, 0.00)
***F. *** ***nucleatum*** **infection**	Intervention	13 (32, 40.62)	2 (18, 11.11)	0 (9, 0.00)
	Control	9 (26, 34.62)	2 (17, 11.76)	1 (8, 12.50)
***T. forsythia*** **infection**	Intervention	9 (32, 28.12)	4 (18, 22.22)	0 (9, 0.00)
	Control	10 (26, 38.46)	6 (17, 35.29)	0 (8, 0.00)

As shown in Table 1, the number of days between the baseline and first follow-up visits were significantly more than those between the first and second follow-up examinations (chi-squared=4.87, P=0.03). As shown in Table 3, the participants in the control arm of the study, on average, took longer to return for the first follow-up visit (P=0.74). On the other hand, in the second period between the first and second follow-up visits, it was the participants in the intervention arm of the study, who, on average, took longer to return for the final study visit (P=0.06). Table 5 shows the hazard for the continued presence of any one of the four bacteria tested for, concerning the data from the follow-up variables. In this table, the hazard ratios for randomization and the plaque scores, though non-significant, were retained in the multivariable model. The hazard for the persistent presence of any one of the four bacteria selected for the study significantly increased with clinical improvement in DASS-28 scores (hazard ratio=1.42, 95% CI: 1.07‒1.89, P=0.01). The comparison of the hazard ratios was significant for the individuals with good response relative to those with no response (hazard ratio=2.18, 95% CI: 1.29‒3.67, P<0.01). There was no difference in the hazard ratio for individuals with moderate and no response (hazard ratio=0.80, 95% CI: 0.47‒1.36, P=0.40), keeping all other factors in the model constant. Each unit increase in the gingival index was associated with a significant increase in the risk of the persistent presence of any one of the four bacteria selected for the study (Hazzard ratio=1.92, 95% CI: 1.11‒3.31, P=0.02). Each unit increase in the pocket depth was, on the other hand, associated with a significant reduction in the risk of the persistent presence of any one of the four bacteria selected for the study (Hazzard ratio=0.61, 95% CI: 0.50‒0.74, P<0.01).

**Table 5:** A summary of the hazard ratios for persistent bacterial presence between the visits

	**Hazard ratio (95% CI, P-value)**	
**Variable**	**Uni-variate model**	**Multivariate model**
**Improvement in DASS-28**	1.15 (0.89 to 1.49, 0.28)	1.42 (1.07 to 1.89, **0.01**)
**Pocket depth**	0.62 (0.51 to 0.76, **<0.01**)	0.61 (0.50 to 0.74, **<0.01**)
**Gingival score**	1.78 (1.07 to 2.96, **0.03**)	1.92 (1.11 to 3.31, **0.02**)
**Plaque score**	0.98 (0.70 to 1.38, 0.91)	0.74 (0.52 to 1.05, 0.09)
**Intervention**	1.00 (0.58 to 1.74, 0.99)	0.92 (0.54 to 1.56, 0.76)

### Oral examination for PD of participants

As shown in Table 1, the baseline median gingival index for the participants was 0.33 (IQR: 0.08‒0.83). There was also no significant difference in the gingival index for the cases (0.51, SD=0.46) and control (0.50, SD=0.51) throughout the study period (difference: 0.09, t-statistic 0.09, P=0.93). Overall, the gingival index reduced with each additional follow-up visit (average difference between visits: -0.30, 95% CI: -0.38 to -0.23, P<0.01). Each unit increase in the gingival index was associated with a 1.13 increase in the DAS-28 score (95% CI: 0.55‒1.71 P<0.01, constant: 4.15 [95% CI: 3.68‒4.64 P<0.01]). The change in the gingival index explained 17% of the variance in the DAS-28 scores for this population (rho=0.17, P<0.01).

As shown in Table 1, at baseline, the median plaque score for the participants was 0.67 (IQR: 0.17‒1.17). There was no significant difference in plaque scores for the cases (0.84, SD=0.52) and controls (0.75, SD=0.51) throughout the study period (difference: 0.09, t-statistics=0.75, P=0.46). Overall, the plaque score reduced with each additional follow-up visit (average difference between visits: -0.40, 95% CI: -0.51 to -0.29, P<0.01). Each unit increase in the plaque score was associated with a 0.76 increase in the DAS-28 score (95% CI: 0.29‒1.21, P<0.01, constant: 4.12 [95% CI: 3.61‒4.64, P<0.01]). The change in plaque score explained 9% of the observed variance in the DAS-28 scores (rho=0.09, P<0.01).

Pocket depth of DPSI >3 was only assessable in 86.70% (65/75) of the follow-up oral examinations. Overall, it was noted that there were 7/65 (10.70%) of previously healthy individuals who presented with observable pockets during the next oral examination visit. Another 38.50% (25/65) showed no improvements, while 50.70% (33/65) had reductions in pocket depth or improvements. There was no significant difference in the pocket depth for comparisons between the treatment and intervention arms of the study (RR=1.00, 95% CI: 0.98‒1.03, P=0.92). The pocket depths decreased with each additional follow-up visit (average difference in the pocket depth between visits: -0.59, 95% CI: -0.79 to -0.39, P<0.01). Each unit increase in the pocket depth was associated with a 0.57 increase in the DAS-28 score (95% CI: 0.23‒0.90, P<0.01, constant: 3.26 [95% CI: 2.26‒4.26, P<0.01]). The change in pocket depth explained 6.00% of the observed variance in the DAS-28 scores (rho= 0.06, P<0.01).

### Overall comparisons and randomization

Table 6 shows that in comparison to the participants who had no DAS-28 score improvements, participants with a moderate improvement in DAS-28 score were 0.61 times more likely to be in the intervention group (RR=0.61, 95% CI: -1.70‒2.91), keeping all the other factors constant. Furthermore, in comparison to the participants who had no DAS-28 score improvement, participants with an overall improvement in DAS-28 score were 0.02 times more likely to be in the intervention group (RR=0.02, 95% CI: -5.43‒5.47), keeping all other factors constant. In this Table, the individuals with a definite improvement were significantly more likely to have much worse gingival indices (RR=24.65, 95% CI: 19.69‒29.62) and more frequently carried *F. nucleatum* bacterial species (RR=29.66, 95% CI: 25.08‒34.24) than individuals with no improvement in DAS-28 scores. Other factors in the model remained non-significant. During the follow-up period, no adverse events were observed by the study team.

**Table 6:** A summary of participant comparisons using DAS-28 Score response

**Variable**	**Uni-variate modeling (95% CI)**	**Multivariable modeling (N, 95% CI)**
**Baseline outcome (no improvement)**		
**Moderate improvement**		
**Age**	0.98 (0.93 to 1.02)	0.97 (0.90 to 1.05)
**Baseline DAS**	2.66 (0.80 to 8.88)	1.61 (0.65 to 2.56)
**Plaque score**	0.89 (0.46 to 1.72)	1.18 (-0.67 to 3.02)
**Gingival index**	0.95 (0.48 to 1.86)	0.60 (-1.04 to 2.24)
***P. *** ***gingivalis***	1.25 (0.25 to 6.22)	2.74 (-0.03 to 5.50)
***F. *** ***nucleatum***	2.66 (0.21 to 34.17)	1.43 (-1.52 to 4.39)
***A.*** ***actinomycetemcomitans***	0.37 (0.06 to 2.17)	1.03 (-1.67 to 3.72)
***T. forsythia***	1.67 (0.31 to 8.99)	2.59 (-0.20 to 5.38)
**Elaborate treatment group**	0.70 (0.23 to 2.13)	0.61 (-1.70 to 2.91)
**Female sex**	0.50 (0.16 to 1.72)	2.84 (0.47 to 5.20)
**Good improvement**		
**Age**	1.01 (0.96 to 1.06)	1.18 (0.88 to 1.48)
**Baseline DAS**	1.81 (0.59 to 5.53)	0.34 (-1.43 to 2.10)
**Plaque score**	1.12 (0.55 to 2.29)	0.14 (-3.30 to 3.59)
**Gingival index**	1.12 (0.54 to 2.34)	24.65 (19.69 to 29.62)
***P. *** ***gingivalis***	0.14 (0.02 to 1.27)	0.0002 (-12.33 to 12.33)
***F. *** ***nucleatum***	6.00 (0.56 to 63.95)	29.66 (25.08 to 34.24)
***A.*** ***actinomycetemcomitans***	1.48 (0.32 to 6.94)	0.02 (-6.76 to 6.79)
***T. forsythia***	0.47 (0.08 to 2.84)	0.02 (-4.91 to 4.96)
**Elaborate treatment group**	0.55 (0.18 to 1.73)	0.02 (-5.43 to 5.47)
**Female sex**	1.50 (0.33 to 6.82)	0.34 (-3.77 to 4.54)

## Discussion

This was a six-month open-label randomized controlled clinical trial in which the participants were followed-up in two 3-month periods. It is the first study to report the impact of treatment of periodontitis in patients with rheumatoid arthritis in our settings where oral care is not part of the routine management of rheumatoid arthritis.

Overall, there was no statistically significant difference between the observed improvements in DAS-28 scores between the two arms of the study. However, there was a significant reduction in the DAS-28 scores in both the intervention and control arms. Similar findings in improvements in the RA disease activity in patients with periodontal disease after intervention for the periodontal disease have been reported previously.^11-14,36^ In contrast to the current findings, no clinical effect of the periodontal treatment on RA was identified in a recent study.^37^ This difference could be explained by the severe disease activity of RA (mean DAS-28 score: 6.75) in the participants recruited in the current study compared to the recent study with moderate disease activity (DAS-28 score: 3.2–5.1). More reduction in the DAS-28 score was noted in the first three months of the current study compared to the next three-month period. This finding is similar to the recent study in which the DAS scores at baseline were comparable to those of the current study after the first three months. However, clinically, the patients from both intervention groups reported that they felt much better with less joint pains and swellings. Probably, within resource-constrained areas with low numbers of dentists, basic oral health treatment should be emphasized and demonstrated to people living with rheumatoid arthritis. Probably, it would be better to further evaluate this by comparing different disease severity groups and recommend the best intervention for each.

Since the improvements in DAS-28 scores between the treatment and control arms were not significant, the subsequent analysis for periodontopathogenic bacteria and pocket depth was carried out together. More than 80.00% of the subjects carried periodontopathogenic bacteria in their pockets at baseline; mixed infection was present in 44.83% of the participants. During the follow-up, there was a statistically significant reduction in the variety of screened bacterial species (chi-squared=23.71, P<0.01). This might be attributed to PD treatment. Different from the current findings, a recent study compared periodontopathogenic bacteria but used counts rather than the presence of the bacteria.^11^ It found that the counts of *P. gingivalis , T. forsythia, and T. denticola* decreased significantly in controls with PD but not in the RA group with PD. Changes in DAS-28 scores correlated positively with those of *P. gingivalis .* However, it did not evaluate the effect of the different species, like the current study.^11^ Since PD is a polymicrobial disease, the effects of other bacteria other than the four studied here are also important. The effects of the other bacteria were shown in a study on the subgingival microbiome and rheumatoid arthritis,^38^ reporting that in the RA patients with active disease, antiinflammatory medication, as part of RA therapy, was associated with better oral health status and a healthier subgingival microbiome compared to that of RA patients in remission, especially those in remission, who were current smokers. However, that study did not examine the effect of periodontal treatment on the microbiome in RA patients. Similar studies have shown that the oral microbiome differs in rheumatoid arthritis as compared to normal controls^39^ and that the oral microbiome is perturbed in rheumatoid arthritis but normalized on treatment.^40^ However, these still examined the whole oral microbiome which is different from the subgingival microbiome, necessitating more studies to examine the effect of the subgingival microbiome as PD treatment is provided for RA patients. Compared to the participants with none of the four bacterial species screened for, those with one or two or more of these bacteria had significantly higher DAS-28 scores (P<0.01).

There was an improvement in the gingival score, periodontal score, and pocket depth between the visits. This reduction in oral parameters of the gingival score, plaque score, and pocket depth has previously been reported.^11,12,37^ Overall, there was an improvement in the RA measure of DAS-28 score with a reduction in the oral parameters of the gingival score, plaque score, and pocket depth, an observation which has also been reported previously concerning another measure of RA SDAI.^12^ In addition, there was no significant difference in the pocket depth between the two study groups. This could be attributed to the inclusion criteria, where we enrolled study participants with DPSI of ≥3. We used the DAS-28 score in this study to measure the RA disease activity, which did not show a statistically significant correlation with the pocket depth. This finding is similar to other studies, although they used different measures for the pocket depth. However, it is still important to emphasize dental hygiene practices to RA patients because impaired dental hygiene is directly connected with periodontitis onset and progression. It is important to note that up to three months of follow-up, both study groups (intervention and control) showed a significant reduction in DAS-28 scores; however, after three months, this reduction was less marked. We postulate that probably lack of re-enforcing OHI among the study participants might have a role in the reduced DAS-28 scores after the first three months.

### Limitations

Only the presence of four periodontopathogenic organisms was studied, which might limit the conclusions we might draw in terms of the effect of counts of the bacteria on DAS-28 scores. Additional information could have been obtained if we had considered counts, too.^11^ This might call for further studies on the subgingival microbiome and on how it affects the DAS-28 scores during these follow-up studies. Additionally, the host factors which might be considered contributing factors need to be studied, and this might be another reason for the differences observed in the improvements in DAS-28 scores after intervention through periodontal treatment reported in the current study and the literature.

## Conclusion

There were improvements in the RA patients’ conditions as measured by changes in the DAS-28 score in the intervention and control arms over the six-month follow-up. A higher number of bacterial types tested for was associated with increased DAS-28 scores. Patients in communities with limited oral health workers could benefit from OHI given to them as additional health care for the management of their RA condition.

## Acknowledgments

We would like to acknowledge Olivia Namusoke and Susan Nakubulwa, who consented to participants and donate the blood samples; Bate Jackson for driving the study team and the participants to the dental clinic; the management of the arthritis clinic at Kiruddu for providing an environment conducive to work in; and the participants who donated their samples.

## Authors’ Contributions

EO conceived the initial research idea, was part of the medical team and was a major contributor to the manuscript. WB, IGM and KM refined the research idea, drafted the initial proposal, and were involved in the whole research process till drafting of the manuscript. WEJ and NKS were instrumental in refining the research methodology, designing the primers, contributed to the drafting of the manuscript and were involved in analyzing the data. WB, MM, HK and EN were part of the dental team, contributed to data collection, and critically read the draft manuscript. All co-authors reviewed the final manuscript prior to submission.

## Conflicts of Interest

The authors declare no competing interests with regards to the authorship and/or publication of this article.

## Funding Statement

This work was supported by a grant [D43TW010132] supported by the Office of the Director, National Institute of Health (OD), National Institute of Dental & Craniofacial Research (NIDCR), National Institute of Neurological Disorders and Stroke (NINDS), National Heart, Lung, and Blood Institute (NHLBI), Fogarty International Center (FIC), and National Institute on Minority of Health and Health Disparities (NIMHD). Its contents are solely the responsibility of the authors and do not necessarily represent the official views of the supporting offices.
